# Weight gain and smoking: perceptions and experiences of obese quitline participants

**DOI:** 10.1186/1471-2458-14-1229

**Published:** 2014-11-27

**Authors:** Terry Bush, Clarissa Hsu, Michele D Levine, Brooke Magnusson, Lyndsay Miles

**Affiliations:** Alere Wellbeing, 999 3rd Ave, Suite 2000, Seattle, WA 98104-1139 USA; Center for Community Health and Evaluation, Group Health Research Institute, 1730 Minor Ave, Suite 1600, Seattle, WA 98101-1404 USA; Western Psychiatric Institute and Clinic, University of Pittsburgh Medical Center, 3811 O’Hara Street, Pittsburgh, PA 15213 USA; Formerly with Alere Wellbeing, 9228 NE 183rd St, Bothell, WA 98011 USA

**Keywords:** Smoking, Obesity, Weight gain, Attitudes, Qualitative

## Abstract

**Background:**

Weight gain that commonly accompanies smoking cessation can undermine a person’s attempt to quit and increase the risk for metabolic disorders. Research indicates that obese smokers have more weight concerns and gain more weight after quitting than non-obese smokers, yet little is known about possible reasons for these outcomes. We sought to gain an understanding of obese smokers’ experiences of quitting and their attitudes and beliefs about the association between smoking and weight gain.

**Methods:**

In-depth semi-structured interviews were conducted with obese smokers who called a state tobacco quitline. Interviewers elicited discussion of obese smokers’ thoughts about smoking, the effects of quitting on change in weight, challenges they faced with quitting, and how quitlines might better serve their needs.

**Results:**

Participants (n = 29) discussed their fear of gaining weight after quitting, their beliefs about smoking and their weight and significant experiences related to quitting. Participants’ awareness of weight gain associated with quitting was based on prior experience or observation of others who quit. Most viewed cessation as their primary goal and discussed other challenges as being more important than their weight, such as managing stress or coping with a chronic health condition. Although weight gain was viewed as less important than quitting, many talked about changes they had made to mitigate the anticipated weight gain.

**Conclusions:**

Weight gain is a concern for obese smokers interested in quitting. Understanding the relative importance of body weight and other challenges related to smoking cessation can help tailor interventions for the specific group of smokers who are obese and interested in smoking cessation.

## Background

Obesity [1, 2] and smoking [3–7] are the leading causes of preventable morbidity and mortality worldwide. Approximately nine million adults smoke and are obese [8–10]. In one study, over two-thirds of candidates for weight loss surgery reported a history of smoking and 27% were smoking at the time of a pre-surgical evaluation [11].

Most smokers gain weight when quitting smoking which can interfere with cessation efforts [12–23]. Although cessation-related weight gain is modest [12, 24–26], many smokers report gaining more than 10 Kg. [13, 19, 22, 24, 27–32], placing them at high risk for obesity-related chronic diseases [25, 33]. Some studies suggest that smokers who are obese before beginning their quit attempt appear to gain an excessive amount of weight (>15 Kg.) after quitting [25, 27], but reasons for this greater weight gain are unclear. Therefore, helping obese smokers quit and avoid or limit weight gain needs to be a public health priority.

While some data exists on the association between baseline weight and weight gain after quitting [27, 30, 34], data on the association between baseline weight and cessation outcomes are sparse and inconclusive. In one quitline study, smokers with high body mass index (BMI) were less likely to quit smoking [35]. Others reported a positive association; higher weight prior to quitting predicted better cessation rates [36] or that baseline weight was not associated with successful quitting [30, 37, 38].

Concerns about cessation-related weight gain are also common and can inhibit a quit attempt and increase the risk for relapse to smoking [19, 36, 39–42]. Having strong concerns about weight gain could be due to experiencing weight gain in prior quit attempts [43] or one’s belief that smoking is an effective method for weight control [20, 44, 45]. Dissatisfaction with one’s weight or body shape has also been shown to be associated with initiation of smoking [44, 46]. Although obese smokers are more likely to be concerned about gaining weight when quitting than are normal-weight or overweight smokers [41, 47], little is known about the source of these concerns and whether the fear of weight gain in treatment-seeking obese smokers would have an impact on treatment outcomes. Surprisingly few studies have explored obese smokers’ attitudes, beliefs, and experiences related to smoking cessation efforts and cessation-related weight gain, information that can be useful in understanding potential barriers to treatment success. To begin to address this gap, we conducted a qualitative study with obese treatment-seeking smokers to explore: (1) how obese smokers articulate the associations between smoking and satisfaction with their weight as well as between quitting and weight gain; (2) whether obese smokers’ attitudes and beliefs provide insights into why this group may gain more weight post-quit compared to non-obese smokers; and (3) whether obese smokers have unique issues that might inhibit a successful quit attempt.

## Methods

Semi-structured, in-depth telephone interviews were conducted with obese smokers. We chose individual interviews rather than focus groups because our study participants were recruited from a geographically dispersed population who were accessing a phone based tobacco cessation service. We followed standard qualitative methods [48]. Participants were asked to talk about their experiences with quitting and thoughts and beliefs about the relationship between smoking, quitting, and weight. Participants who completed the interview received a $30 gift card for participating, were encouraged to continue with the quitline counseling calls, and to call the quitline for additional support as needed. The study and all protocols were approved by the Western Institutional Review Board (July 1, 2009).

### Setting - recruitment

Participants were recruited through the Georgia and South Carolina quitlines. Our target sample size was based on qualitative research designs, the diversity of the sample, and our experience which indicated that we needed 25–30 participants to ensure that we captured multiple perspectives on all the topics of interest. We stopped recruitment at 29 individuals because we had sufficient participation across all the key variables in our samples.

### Interviews – participants and data collection

Study participants were tobacco users who called the state quitline between November 2009 and March 2010 and completed the standard quitline assessment questions plus two study questions about height and weight. Eligible callers were invited to take part in the study if they met the following criteria: smoke at least 5 cigarettes per day; not pregnant or nursing; age 18 years or older; speak and understand English; ready to quit within 30 days; have a body mass index (BMI) > =30; provided their race and/or ethnicity and an address and phone number where they could be reached. Sampling targets were set to ensure interviews were conducted with individuals who generally mirrored the ethnic and gender distributions of the overall quitline population [47], which are 40% male and 30% African American. Eligible callers who provided verbal informed consent were mailed a letter explaining the study, and were then contacted by phone for an in-depth telephone interview. Once sampling targets had been met for each demographic, participants in that demographic were no longer recruited. Interviews occurred within 19 days of participant’s first counseling call.

### Procedures

The study team developed a semi-structured interview guide consisting of open-ended questions to elicit obese smokers’ thoughts and beliefs about smoking cessation, body weight, and weight gain. The interview guide was based on expert advice, prior research and pilot data, and constructs known to be related to cessation. Participants were first asked two questions to assess weight concerns [20]: (Q1) *“on a scale of 0–100 with 100 being very concerned and 0 being not concerned at all, how concerned are you about gaining weight after quitting,?*” and (Q2) “*how concerned would you be if quitting smoking caused you to permanently gain 10 pounds*?” Interviewers then probed for discussion of participants’ motivation to quit, previous and current quit experiences, exercise and eating habits, and prior use of weight management programs. Participants were encouraged to freely discuss their opinions. Two members of the research team (first 2 authors conducted the telephone interviews). Interviews lasted 20–60 minutes in length and were audio recorded and transcribed.

### Data analysis

Interviews were analyzed using an immersion/crystallization approach [49]. A code list was developed from themes that emerged based on a deep knowledge of the data (conducting interviews and reviewing the transcripts) as well as on the key concepts used to develop the interview guide. The code list was refined through an iterative process in which two or more members of the team coded a transcript and then compared coding results, recommended additions or changes to the codes, and clarified definitions of codes. After three rounds of comparing coding, the team felt that sufficient consensus had been reached to independently code the remaining transcripts. Examples of these codes include “concern about gaining weight,” “chronic disease,” and “satisfaction with one’s weight.” One team member served as the primary coder (2^nd^ author). Half of the transcripts were also coded by other team members and then reviewed by the primary coder. The primary coder finalized all coding and documented the codes using Atlas.ti. Output from this first round of coding was queried, reviewed, and a second round of coding was conducted to identify more specific themes within the data.

## Results

Table 1 shows the characteristics of the 29 study participants. BMI ranged from 30–48; most (86%) were heavily addicted (first cigarette within 30 minutes of waking), 72% had been smoking for 20 or more years and over half (59%) reported having one or more chronic disease (chronic obstructive pulmonary disease, coronary artery disease, diabetes or asthma). With one exception, all participants had made at least one prior quit attempt and 82.7% were classified as having concerns about cessation-related weight gain. Qualitative analyses revealed a number of themes around the following issues: 1-current body image and tolerance for gaining weight; 2-weight concerns and balancing weight gain against benefits of quitting; 3-perspectives on the connection between cessation and weight gain.

**Table 1:** **Demographic characteristics of participants (N = 29)**

Age mean (range)	44.3 (26–64) years
**BMI (30–48)**	**N=**
30-34.9	16
35-39.9	11
40-48	2
**Gender**	**N=**
Male	11
Female	18
**Education:**	**N=**
Less than grade 9	4
Grade 9–11, no degree	3
High School Degree	10
Genera education degree	4
Some College or University	4
Technical/Trade Degree	1
College/ University Degree	2
**Race/ethnicity**	**N=**
African American	9
White	19
Other	1
Non-Hispanic	29
**Uninsured**	14
**Chronic disease** (asthma, chronic obstructive pulmonary disease, heart disease, diabetes)	**N =** 17
**Duration smoked**	**N=**
6-19 years	8
20+ years	21
**Time to first cigarette**	**N=**
5 minutes	14
6-30 minutes	11
31-60 minutes	2
>60 minutes	2
**Number prior quit attempts:**	**N=**
0	1
1	6
2-5	13
6+	8
**Confidence in quitting** mean (s.d) (scale = 1-10);	8.0 (2.07)
Number scoring 6+	18
**Scored 50+ on weight**	**N=**
**concerns scale (0–100)**	24

### Body image and factors affecting views on cessation-related weight gain

Most participants (21 of 29) expressed dissatisfaction with their current or past size or shape; “*It’s bad for your health to gain weight and it looks bad”;* “*I would hate to look fat again”* or “*I’m not very satisfied with my weight right now, me and my fiancée are trying to lose weight.”* Participants were conscious of their weight but many had built mechanisms to cope with being overweight, for example one participant noted: *“I look at it this way. If they don’t like who I am or my size, then they don’t have to look.”* As many as 16 participants were actively trying to control their weight; nine said they were eating healthier foods but were not restricting the amount of food or counting calories and *s*even stated that they were *“on a diet.”* This seemed to imply they were following a structured eating regime or restricting their food or calorie intake to lose weight or avoid weight gain; *“I don’t do strict diets, just a regular diet”* and one person stated, *“I am starting to eat less food, use a small plate”.* At least five participants did not expect their weight would change much after quitting: *“I don’t think that the weight gain will be too tremendous. I just feel like I can really do it”* (quit smoking), while others seemed resigned to being overweight: “I will *never be skinny or have a flat belly like these young people do or I’ll never look good in a bikini*.”

Of the17 participants who reported having a smoking or obesity-related chronic health condition, these individuals recognized that weight gain further impacts their health. One participant noted these concerns: *“This adds back the weight I had before I had diabetes,”* and another said: “*My two biggest fears are weight gain and COPD. The fat comes with me when I stop smoking, and the COPD comes with me smoking. So it’s like, I’m damned if I do, and I’m damned if I don’t. But I’m making’ myself happy if I stop smoking.”*

At least three participants mentioned conversations they had with healthcare professionals who talked about the necessity of losing a significant amount of weight: “*doctors are now telling me I need to lose at least 50 pounds. The last time I was weighed, I was 230 pounds and now that sort of made me feel bad.”* Others reported receiving similar advice about tobacco as one participant stated; “*She was blunt with me and said you’ve got to quit; she said she can’t do anything more for my breathing.”* While over half of participants mentioned that cessation was clearly related to weight gain, a common sentiment was that weight gain was the better of two evils when compared to smoking, suggesting some tolerance for weight gain: *“….gaining weight and not smoking is more important. If it means that I have to like run a mile, I’ll run a mile.”* Having a chronic disease and worries about long term health effects of both smoking and obesity appear to provide important external motivations for participants to change their behaviors (e.g. doctors’ advice about smoking and/or their weight).

### Weight concerns -balancing weight gain against benefits of quitting

Concerns about their current weight in general were clearly an important factor affecting individual’s apprehension about weight gain associated with smoking cessation. 27 participants were asked whether they were concerned about gaining weight during the current quit attempt. Of these, 11 said they had concerns, 10 said they were not really concerned and 6 provided mixed or unclear responses. For the latter 6 participants, their discussions of weight concerns were ambiguous or contradictory. These unclear responses may highlight the difficulties obese smokers face when trying to make positive lifestyle changes in light of their conflicted feelings about their weight, a lack of control over their eating and smoking and about how concerned they should be about their weight.

Regarding those with significant concerns about weight gain, these individuals talked in general terms of their desire not to gain weight: “*I’m scared to death. I don’t want to be fat”* or: *“I don’t want to gain the weight because it’s not good for my health and also I want to look better for myself”* or the effect weight gain has on one’s mood and general wellbeing: *“Just gaining weight and irritability* (…due to quitting*), that’s all that I’m worried about.”* Participants also expressed a strong fear or anxiety about gaining additional weight, including the participant who shared that: “*It kind of scares me because right now, I’m overweight pretty bad. I’m about 200 pounds now and I want to be 135. So it’s kind of worrying me but I know if I keep exercising even when I do stop smoking, it will help with the weight gain.”* Sometimes excessive weight concerns and intolerance for any weight gain results in a smoker working on two difficult health behaviors at once: *“I really don’t want to gain more weight and I really want to quit smoking, so I am trying to do both of them at the same time.”*

These statements seem to emphasize the conflicts and contradictions obese smokers have about their current weight and quitting smoking. It is a catch-22 situation whereby they are concerned if they don’t quit because they are endangering their health by smoking and they are concerned if they gain weight while quitting because of the health effects of weight gain.

Despite dissatisfaction with their current weight, a substantial number of participants did not appear concerned about weight gain or stated that their worry over weight gain would not interfere with their commitment to quit smoking. Only one person mentioned that he would go back to smoking if he gained weight: *“I think I would be more at risk going back to smoking if there was another family tragedy and me gaining weight”.* Participants seemed hopeful they would be able to manage cessation-related weight gain or expressed a relative lack of its importance compared with other challenges they were facing: “*Smoking is the most important to me; as far as weight gain, yeah, I’m real concerned but you just got to watch yourself; you can’t take one addiction for another. If one’s not going to kill you, the other will, more or less, so it’s not something that a person has a choice about.”* Another participant noted: *“I know the biggest thing in my house is getting rid of cigarettes because of depression. As far as gaining weight, I’m going to gain it or lose it; either way it’s not really concerning. I’m to a point where I’m tired of smoking, its 5 or 10dollars every other day, or every day whatever you spend on cigarettes. So I just got to quit.”* Similarly this participant noted: “*Oh I know I would gain weight, there’s no doubt. But I’m not worried about it because the weight, I can lose it by walking.”*

This participant illustrates a common theme of an awareness of the potential for weight gain, but a strong commitment to prevent that weight gain: *“I just don’t see myself gaining weight, I just won’t allow it. If I take care of myself and do the exercising and the walking and just watching what I eat, then it won’t be an issue. I mean I would hate it but it’s not going to be that much of a deterrence to make me start smoking again just so I won’t gain the weight.* Similarly two other participants noted: *“I’m a little concerned about it because I don’t want to be any heavier than I already am but that’s not going to stop me from quitting. I’m trying to lose weight.”* and *“I’m not worried about gaining 5or 10 pounds to quit. Even if it was more, I’ll start walking more, so I think I’m going to lose it.”* These quotes also highlight the awareness or expectation of weight gain and an understanding among a number of participants that increasing physical activity was an important factor in maintaining their weight. However, physical activity was not considered for those with mobility limitations possibly due to their smoking and/or weight*; “I can’t walk even 1block or I get out of breath” or “I got no knees, I mean I have really bad knees and can’t walk”.*

About half of the participants were conflicted about where to focus their energies; to work on quitting smoking, losing weight or addressing other more concerning issues such as dealing with chronic disease and/or managing stressful life situations: *“I have 4 stents in my heart; I’d like to stay around a little longer”* and *“Stress and time interferes with eating better and my ability to quit. When I have no time to eat it gets to a point I need my cigarette.”* Such sentiments reflect the potentially unique challenges obese smokers may face as they attend to multiple smoking and/or obesity related health issues all requiring difficult and sometimes conflicting lifestyle change.

Time constraints were also apparent challenges for this long haul truck driver who described the stress of both quitting smoking and maintaining his weight while he was on the job: “*I won’t have time to sit down and eat like I should. Shoot, I just buy a pack of cigarettes and I run, run, run, run. And I make stops to grab a hotdog and I get back in the truck and I run, run, run, run. You don’t think about it, you’re just smokin’ and rollin”.* Others noted that they were more concerned with managing their anger and irritability during the quit process than worrying about their weight: “*I’m not so much worried about gaining weight when I quit smoking. I’m worried about hurting somebody. I’m worried about being so stressed out that, if I don’t have a cigarette to go smoke, you know, what do I do then?”* Such strong statements of the addictive properties of smoking are not uncommon as indicated by this person; *“You’re addicted to it. I have tried to go cold turkey. It does not work, I just go berserk. It’s like someone being on a different type of drug. You’ve just got to get that money, you’ve got to get that cigarette; you have got to have it. I never thought anything would take control of me, but, yes, cigarettes have.”*

### Perceived connection between cessation and weight gain

Expanding on this subject, participants were asked to discuss their beliefs, experiences, or expectations regarding the potential for, and magnitude of weight gain associated with cessation. Overall, participants expected to gain weight during the cessation process: “*I know I’ll gain weight. I expect to gain 10–15 pounds because I’ll have a better appetite.”* This participant had already experienced cessation-related weight gain, and had concerns that this would occur again. “I *gained 20 pounds within 2 weeks after quitting smoking and it was terrible.”* Others shared experiences of substantial weight gain: *“The last time I quit, I was 140 pounds I think, but I reached 201 pounds.”* Similarly, this participant noted: “*I was at 228 [pounds], and I’m back up to 252. I know it was related to quitting because I was snacking on everything.”*

Individuals did not necessarily associate all of their weight gain with quitting. This person half-jokingly explained that quitting smoking provided a socially acceptable explanation for recent weight gain due to a change in work environment and holiday overindulgence: “*It’s hard because during Thanksgiving and Christmas, I really picked up some weight. I thought “Ok. I need something so when I finally do go out and see friends and people that I work for; I need to have an excuse for gaining so much weight. I’ll tell them I quit smoking.”* This person had started working from home and found that he ate more and exercised less. His insight illustrates the unique and complex thought processes of obese smokers.

As we see above, a number of smokers reported personally experiencing weight gain that they associated with smoking cessation. However, there were a number of other sources of information about this issue. Participants mentioned that they had heard about weight gain from others or observed weight gain among people who had quit: “*My cousins have already talked about it and that’s why they are afraid of gaining weight”* or *“I heard that’s one thing that happens when you quit because the oral thing and you’re sticking things in your mouth, so it’s usually food”* and *“I didn’t want to get fat. I’ve seen what it did to a lot of my friends, and they got obese, and oh, God, I couldn’t do it.”*

Still others saw no association between smoking, quitting, and changes in weight. Instead, they observed that *“A lot of people smoke and yet they keep getting bigger.”* This sentiment was shared by another participant, who emphasized that while people may gain weight after cessation, many of them started the process already overweight: “*I don’t think smoking has anything to do with it because you got a lot of people that smoke that are big and they KEEP getting bigger but they keep smoking. I had a weight problem when I WAS smoking.”* Another specifically states that food intake rather than smoking is the primary reason for their weight gain: “*I know it doesn’t have anything to do with smoking. It’s those little cheesecakes.”*

Participants appeared to be aware of the impact that smoking and quitting could have on their appetite and/or eating habits. At least three individuals identified smoking as a means for maintaining or losing weight; “*I know that the only positive benefit from smoking at all is that it will cause people to lose weight. It raises your metabolism.”* One participant stated that their *“eating habits changed a lot”* when they were smoking (e.g., lost their appetite) and changed again when they quit smoking (increased craving for food, especially snacks). “*You replace eating for smoking;” and “you know, as a smoker, when I get hungry? I want to smoke. You’ve got the hand-to-mouth motion.”* Participants shared their concerns about increased appetite and increased cravings for certain foods after quitting, especially snacks; “*It seems like I want to eat more, but not because I’m hungry. I just want to eat.”* Smokers also mentioned that food tasted better after quitting. “*I know when you stop smoking everything tastes better. And so I’ve got that fear that I’m going to pile it back on.”*

Participants reported that smoking can be a substitute for eating but smoking can also become an integral part of meals or eating rituals. “*Any smoker will tell you that when you’re done eating, the cigarette is kind of the dessert, to finish off the meal. It lets your mind know, ‘Hey, I’m done eating.”* Such strong connections make it difficult to disassociate these two behaviors (smoking and eating). In fact the behavioral connection between finishing a meal and having a cigarette was noted as an important obstacle to face while quitting smoking. One person shared that “*one of the big things I’m working on right now is that when I’m done eating, to NOT smoke. I’m trying’ to break myself of smoking right after I get through eating.”* Others tried to substitute food for cigarettes in order to quit smoking. In some cases, these strategies proved to be counter-productive: “*Sometimes I eat when I’m trying to stop smoking and I’ll eat more. But I’ll choose a cigarette over food in a minute. But then later on I’ll come back and eat anyway.”* The ineffectiveness of this strategy was further noted by another participant: “*I know when I don’t eat, I’ll want something to eat, and rather than eat, I say, ‘Ah, I’ll smoke a cigarette’. Now, when I say, ‘I’m not going to smoke, I’m not going to smoke’ then I go to look for something to snack on. Then I know if I snack I’m going to gain weight.”* As we see, obese smokers’ concerns, while present, have as much to do with balancing the need to control both weight and smoking as they do with weight *gain*.

## Discussion

In summary, although there was variability across our sample, the following themes were most commonly expressed by the obese smokers who called a quitline: dissatisfaction with one’s weight and size; a recognition of the relationship between cessation and weight gain, tolerance for weight gain, importance of past experiences with cessation and the challenges of managing stress and other chronic diseases.

Clearly, the struggle to quit smoking is complex, and for a lot of people, the actual or feared weight gain further complicates treatment. To the best of our knowledge, this paper is the first qualitative study to explore the ways obese smokers articulate their experiences and challenges with quitting smoking. A surprising finding was the number of obese smokers who did not express concerns about weight gain. Instead, they appear to recognize the need to balance their potential for gaining weight with the health benefits of quitting smoking. Relapse to smoking if they gained weight also did not appear to be a concern for obese smokers. However, a substantial proportion of participants acknowledged that smoking cessation was often associated with increased appetite and changes in their eating habits and talked about their attempts to control their weight through diet and exercise. Importantly, some studies have shown that restrictive dieting while attempting to quit smoking can undermine the quit attempt [20, 50, 51].

While there are evidence-based interventions that successfully address smoking cessation and weight management treatment (simultaneously or sequentially) [28, 52–54] , these types of interventions are not widely used. One successful approach for smokers who are overly concerned about weight gain might be the cognitive behavioral strategy to address smokers beliefs or concerns about cessation and weight gain which was proven effective for in-person and phone based cessation programs [20, 47].

Also uncovered in these interviews was the view of obese smokers about their current body weight and/or shape. Eliciting individuals’ beliefs about their body image can provide a venue for discussing the struggles they may be facing related to cessation and weight gain. This is important because research indicates an association between body image and cessation efforts [16, 43, 55]. In one study, individuals who received counseling on improving their body image had better quit rates and less weight gain compared with those who received a physical activity intervention [56]. Although these studies were primarily conduced with women or special populations, most of whom were not obese, the obese smokers who do endorse similar concerns about their current weight or shape and the potential to gain more may benefit from such strategies. Indeed, normalizing cessation-related weight gain and preparing smokers regardless of their current weight to accept moderate weight gain rather than return to smoking might be a potent addition to quitline counseling [24].

Both smoking and obesity are challenging health issues that are embedded in complex personal and social contexts. While participants expressed positive statements about their ability to quit and lose or maintain their weight, they also acknowledged the many difficulties they faced. Although it is still not clear what the best treatment is for obese smokers regardless of their weight concerns, topics discussed in these interviews might be useful for tailoring cessation treatment. For example, the participant who described her intolerance for gaining weight because she could not afford to buy more clothes might benefit from discussions about how much money she will save when she no longer buys cigarettes. Individuals who are already on restrictive diets might benefit from a discussion of physical activity and healthy eating strategies to avoid drastic reduction in calories. Providing the skills and support to build one’s confidence in avoiding relapse after quitting are likely to be well received by obese smokers. Incorporating stress management into smoking cessation counseling to help individuals anticipate and deal with stressful life circumstances without turning to unhealthy habits (over eating or smoking) is an essential part of quitline counseling. In fact, research indicates that life circumstances pose a greater barrier to quitting and avoiding relapse than symptoms of nicotine withdrawal [57]. Clearly, individuals in our study were balancing numerous challenges and their perspectives and behaviors may be adaptive given the complexity of issues they are dealing with. Having a weight-related chronic disease could affect one’s tolerance for weight gain and how much weight gain they would accept, and/or their a confidence in not gaining weight because of their chronic disease: *“I don’t much worry about gaining any weight because I know I can’t gain it so much with the diabetes, so I don’t think I’ll go overboard with that”.*

It is important to note potential study limitations. All of the participants had made a significant commitment to quitting by calling the quitline. In the U.S. over 70% of smokers want to quit smoking but less than 6% call the free nationwide quitline for help. Participants in our sample appeared willing to accept some weight gain in order to achieve their goal. In addition, regional and cultural issues may differ from other populations. Participants were from Georgia and South Carolina, nearly half had limited health insurance, and only a minority was educated beyond high school. Since participants in this study were in the early stages of attempting to quit smoking, it is unlikely they would be experiencing the effects of the absence of nicotine and the routine of smoking. Thus, confidence in their ability to handle possible weight gain might change once a participant had quit for some time and experienced withdrawal symptoms or weight gain. Finally, BMI was based on self-reported height and weight collected prior to being told about the study. It is possible that individuals misreported their weight. However, studies have shown strong correlations between measured and self-reported weight indicating that self-reported weight is an excellent approximation of actual weight across a population [58, 59].

## Conclusions

In conclusion, this study provides important qualitative information from quitline participants who are obese and serves to better understand dimensions and complexity of the quitting experience specific to this population. A menu of intervention strategies are needed to help smokers disentangle the relationships between eating, weight management and smoking in order to benefit fully from behavior change interventions such as quitlines.

## Abbreviations

BMI: Body Mass Index
Kg.: Kilogram
COPD: Chronic Obstructive Pulmonary Disease.
